# The Impact of Parental Stress on Italian Adolescents’ Internalizing Symptoms during the COVID-19 Pandemic: A Longitudinal Study

**DOI:** 10.3390/ijerph18158074

**Published:** 2021-07-30

**Authors:** Ziqin Liang, Claudia Mazzeschi, Elisa Delvecchio

**Affiliations:** Department of Philosophy, Social Sciences and Education, University of Perugia, Piazza Ermini 1, 06123 Perugia, Italy; ziqin.liang@studenti.unipg.it (Z.L.); claudia.mazzeschi@unipg.it (C.M.)

**Keywords:** anxiety, depression, parental stress, expressive suppression, adolescent, COVID-19

## Abstract

The challenges and consequences of COVID-19 imposed massive changes in adolescents’ daily routines (e.g., school closures, home confinement, and social distancing rules), which impacted their mental health. This longitudinal study aimed to better understand the changes in adolescents’ internalizing symptoms and the underlying mechanisms of parental stress due to COVID-19. We asked 1053 parents of adolescents to complete an online survey during the second and fifth weeks and at the end of home confinement (i.e., four weeks later). Results showed that parents reported their adolescents’ anxiety and depression symptoms were more severe at Time 2 than at the first administration. Anxiety symptoms slightly decreased at Time 3, while there was no significant change in depression symptoms. Moreover, parents’ expressive suppression mediated the association between parental stress and adolescents’ anxiety and depression symptoms, respectively. The findings suggest that as restriction increased, adolescents’ anxiety and depression became more severe. Moreover, due to the link between parental stress and adolescents’ internalizing disorders helping families to cope with the distress due to the pandemic may have a positive impact on parents, the child, and the family as a whole (i.e., the family climate).

## 1. Introduction

Due to COVID-19, a strict lockdown was imposed across Italy from March 2020, and schools were suspended. To this day, in some countries like Italy, adolescents have not returned to school as normal. Under the strict home confinement rules, adolescents were restricted from learning and outdoor activities, and this impacted their mental health. Previous studies have shown that COVID-19 would cause emotional and behavioral problems [1], and quarantine is related to long-term psychological problems, such as stress, depression, and anxiety symptoms [2]. Adolescents are likely to show long-term psychological problems, such as generalized anxiety disorders after being in this state for a long time [3,4]. The Secretary-General of the United Nations stated, “the crisis is far from over” [5]. It is crucial to establish the changing trend of adolescents’ internalizing symptoms during the pandemic and the home confinement period, as well as the related mechanisms of the influencing factors. However, to date, few studies exist on these issues. To bridge these gaps, the first aim of the current study was to examine the adolescents’ internalizing symptoms during the COVID-19 quarantine. The second aim was to examine the predictive effect of parental stress on adolescents’ internalizing symptoms and how parents’ expressive suppression could potentially explain how parental stress predicts adolescents’ internalizing symptoms due to its relationship to internalizing symptoms and its role as emotion regulation strategies in times of distress. We hypothesized that higher parental stress would predict increased adolescents’ internalizing symptoms via increased parent’s expressive suppression.

### 1.1. Adolescents’ Internalizing Symptoms during COVID-19 Quarantine

Adolescence is often considered a period of storms and stress [6]. Terranova et al. [7] suggested that adolescents face a greater risk during major events due to the possible interaction between normal adolescence-related distress and their more accurate, although not yet fully mature, perception of the seriousness of the situation. The COVID-19 epidemic is a global public health emergency. It not only brings the threat of death, but the strict home confinement rules disrupt the normal routines of adolescents’ lives, and the subsequent stressful events may lead to increased mental health issues [1,2,8,9]. Recent longitudinal studies have compared the mental health status of adolescents during COVID-19 with that before the pandemic, and these showed that COVID-19 has caused a sharp increase in the adverse psychological adjustment of adolescents [10,11,12,13]. For instance, Li et al. [10] investigated the mental health of Chinese college students during the initial stage of COVID-19 (before quarantine) and during the quarantine period and found that increased negative effects, anxiety, and depression symptoms were observed after two weeks of confinement. Similarly, Magson et al. [13] found that, compared with the states before the COVID-19 outbreak, after two months following the implementation of government restrictions and online learning, adolescents’ depressive symptoms and anxiety increased significantly, and increased conflicts with their parents during the period predicted increases in mental health problems. Fruehwirth et al. [12] conducted a survey of first-year college students in the United States and found that the pandemic led to an increase in the prevalence of anxiety and depression.

However, few studies have examined information on longitudinal changes in adolescents’ mental health during the COVID-19 pandemic. In a recent longitudinal study, Wang et al. [14] investigated the mental health of the general population after the initial outbreak of COVID-19 and four weeks after the outbreak in China. They found no significant longitudinal changes in the levels of stress, anxiety, and depression in these two surveys, but young people and students (12 to 21.4 years old) reported a higher psychological impact of COVID-19 in the second survey. Fernández-Abascal and Martín-Díaz [15] evaluated the mental health of Spanish undergraduates before and during the COVID-19 pandemic (in an ordinary week, one week before the establishment of confinement, and after several weeks of confinement) through a longitudinal study. They found that participants’ positive states gradually decreased over time, the negative effects remained stable, and depression levels remained stable over time. The existing cross-sectional studies on COVID-19 and the mental health of adolescents are not sufficient to describe the longitudinal changes. Adolescence represents a sensitive period of increased depression and anxiety [16]. Therefore, it is important to pay attention to adolescents’ mental health at this special stage and especially to changes in internalizing symptoms.

### 1.2. Parental Stress and Adolescents’ Internalizing Symptoms

Parental stress refers to parents’ feelings and thoughts on stressful situations directly related to them and measures the degree of unpredictable, uncontrollable, and overloaded events in their lives [17,18]. The outbreak of the COVID-19 pandemic and its restrictions has become the most important stressful event for parents recently and has resulted in challenges for families and work [19]. Previous studies have shown that parents perceive high levels of stress, anxiety, depression, and other negative emotions due to the COVID-19 [9,20,21].

During the COVID-19 pandemic and its restrictions, adolescents had to spend more time with their parents than usual, and the parental impact on their children is particularly important. Previous studies on major disasters or public health emergencies emphasized that parents can exacerbate or buffer the impact of stressful experiences on adolescents in times of crisis [22,23,24]. Parental active support (for example, active parent–child discussions) can alleviate children’s and adolescents’ psychological problems in particularly stressful situations [25,26]. However, parents with higher levels of stress and poor mental health may be risk factors for children’s and adolescents’ adaptation following disasters [27,28]. Previous studies showed that COVID-19 brings extra stress to parents, which may increase the risk to children and adolescents of emotional and behavioral problems [29]. Lorenzo et al. [30] investigated the level of internalizing symptoms of adolescents and their parents during COVID-19 quarantine through a longitudinal study and found that a higher level of parental internalizing symptoms would predict a higher level of internalizing symptoms in adolescents in the subsequent month. A transcultural study in samples from Italy, Spain, and Portugal found positive associations between levels of parental stress due to COVID-19 restrictions and internalizing symptoms in their children [31]. Spinelli et al. [32] also found that parental stress was associated with children’s psychological problems; i.e., parents’ perceptions of difficulties associated with COVID-19 home confinement increased parents’ dyadic parenting stress levels, which in turn increased children’s emotional and behavioral difficulties. Parents are adolescents’ closest companions with the most contact during the COVID-19 quarantine, and parental stress has a particularly prominent impact on the internalizing symptoms of adolescents. Establishing the influence mechanisms can help parents find ways to support their children and prevent them from experiencing severe internalizing symptoms.

### 1.3. The Mediation Role of Parents’ Expressive Suppression

Negative emotions (such as stress, anxiety, and depression) are common during COVID-19 quarantine, and emotion regulation is important [19,33]. According to the emotion regulation model proposed by Gross [34,35], cognitive reappraisal and expressive suppression are the two major emotion regulation strategies. Expressive suppression refers to the inhibition of ongoing positive or negative emotional expression behavior [34,36]. In the current study, we focused on expressive suppression. In the first instance, expressive suppression is a form of a response-focused strategy used to regulate emotions after the emotional tendencies have been generated [34,35,37]. Second, expressive suppression is more related to internalizing symptoms (such as anxiety and depression) than cognitive reappraisal [37]. Moreover, previous studies have shown that COVID-19 health anxiety may prompt individuals to suppress their emotions [38]. In the face of stress and threats (such as the COVID-19 epidemic), expression suppression may be a particularly relevant candidate, with a role in the association between parental stress and their children’s anxiety and depression symptoms.

Gross [34,37] states that individuals who are accustomed to suppressing expression often deal with stressful situations by concealing their inner feelings and suppressing external emotions. They will think about the events that make them feel bad in a less favorable or accepting way; they experience more negative emotions than those who use suppression less. Some studies show that when facing a state of high-stress, if individuals use expressive suppression more frequently, they tend to feel burnout [39], and this aggravates their negative feelings [35]. Moore et al. [40] found that expressive suppression is often positively correlated with stress-related symptoms, such as post-traumatic stress disorder, anxiety, and depression. Yeung et al. [41] also found that younger workers who habitually use expressive suppression will experience more work-related stress than their peers who use expressive suppression less frequently. In a recent study, Jiang et al. [42] investigated the impact of the COVID-19 epidemic on post-traumatic stress (PTS) symptoms and emotional regulation of Chinese people, and the results showed that participants with greater PTS symptoms tended to use expressive suppression more frequently. In this sense, in the face of the high stress due to COVID-19, parents may be likely to use expressive suppression as an emotion regulation strategy.

Observing and modeling, parenting practices, and the family’s emotional climate all affect children and adolescents’ emotion regulation, social competence, and problem behaviors (such as internalizing and externalizing) [43,44]. One of the important mechanisms is that by observing their parents’ emotional expression, children and adolescents learn about emotions and emotion regulation [43]. Emotion regulation models also emphasize that if parents suppress their own emotional expressions (for example, unsupportive responses to a child’s maladjustment), it may become the child’s social reference, causing them to adopt their parents’ emotion regulation strategies as their own [36,37]). Suppression of adolescents’ negative emotions may affect their emotion regulation and social functions, the most common of which causes high internalizing symptoms [36,45,46]. For instance, Rogers et al. [47] found that mothers’ use of suppression strategies may convey to their children that their children’s emotions are inappropriate or unworthy, resulting in the children suppressing their own emotions and developing emotional instability. Perry et al. [48] also found that mothers’ non-supportive responses (e.g., dismiss and minimize negative emotions) to the child’s early negative emotions can lead to poor emotion regulation in the middle of their childhood, and this poor emotion regulation increases poor physical and behavioral regulation (e.g., more internalizing symptoms and risk-taking behaviors) during their adolescence. Due to the home quarantine and social distancing rules during COVID-19, adolescents mainly adjust themselves according to their parents’ emotions and reactions [49]. Parents’ emotion regulation strategies have become the adolescents’ main social reference. In this sense, parents’ expressive suppression may predict adolescents’ internalizing symptoms.

In addition, emotional suppression may inhibit an individuals’ social function and reduce interpersonal skills [34]. Trougakos et al. [38] showed that individuals with high levels of anxiety about COVID-19 lacked psychological need fulfillment due to emotional suppression, thereby reducing their engagement with family. In this sense, parents with high levels of stress may ignore their child’s emotional state. At the same time, external stressful life events (such as COVID-19) may directly increase parental incompetence, conflict, or frustration, which may increase the child’s internalizing symptoms [22]. For instance, a study by Spinelli et al. [50] showed that parents with more stress due to COVID-19 were less involved in their children’s activities, thereby reducing their children’s effective emotion regulation. Achterberg et al. [51] found that the increased stress due to COVID-19 could lead to negative parenting behaviors, which increased the child’s stress experience. Emotion regulation theory and previous studies have shown that parents experience higher stress levels than before due to COVID-19 and its restrictions, and in this negative emotional state, parents may use expressive suppression as the main emotion regulation strategy more frequently. However, this suppressive emotion regulation strategy would have a negative impact on their children’s emotions and internalizing symptoms. Taken together, we hypothesized that parents’ expressive suppression is likely to mediate the association between parental stress and adolescents’ internalizing symptoms (anxiety and depression).

### 1.4. The Current Study

This study explored the longitudinal changes and influence mechanisms of adolescents’ internalizing symptoms (anxiety and depression) during the COVID-19 pandemic and its restrictions to provide a theoretical basis for the recovery of adolescents’ mental health and to help prepare for the forthcoming global health emergency. Specifically, we hypothesize that: (1) adolescents’ anxiety and depression symptoms would increase over time, (2) higher levels of parental stress would predict an increase in adolescents’ anxiety and depression symptoms, and (3) parents’ expressive suppression would mediate the association between parental stress and adolescents’ internalizing symptoms.

## 2. Materials and Methods

### 2.1. Participants and Procedures

A total of 1053 Italian parents (*M* = 46.43, *SD* = 5.45, 88.2% mothers) of adolescents aged 11–18 years old (*M* = 14.13, *SD* = 2.25, 49.1% girls) participated in one of three longitudinal online surveys. A total of 452 (attrition rate = 57.1%) and 288 (attrition rate = 36.3%) parents participated at times 2 and 3 (T2 and T3), respectively. Table 1 lists the sample characteristics. In summary, 99.7% of participants (*n* = 288) were Italian nationals, and 76.8% had more than one child. Of the participants, 75.3% had a monthly family income of more than 2000 Euros, 46.5% of parents held a bachelor’s degree or above, suggesting that most of the families came from a middle-class context (i.e., SES level 3; [52]). Of the parents, 78.5% of mothers and 94.2% of fathers had a job, and only 13.5% of mothers and 5.8% of fathers reported that they were unemployed or had lost their job due to COVID-19.

The current study was conducted in compliance with the guidelines reported in the Declaration of Helsinki and was approved by the Ethical Committee for psychological research of the corresponding author’s university. Due to COVID-19 and its restrictions, a snowball sampling strategy was used to recruit participants on social networks (such as WhatsApp groups) and/or emails to join in the study, and participants completed the surveys through an online platform. Before they completed all the questions, the participants were notified of the basic study information, and informed consent was obtained. Inclusion criteria for participation were: (a) 18 years old or over, (b) having one or more children aged 11–18 years old, and (c) living in Italy. Only one parent of each family was required to participate, and they were asked to complete a survey for each child. Data were collected at three time points. At Time 1 (T1, two weeks after home confinement, March 2020), parents completed the sociodemographic information and reported their child’s anxiety and depression symptoms in response to the quarantine. At Time 2 (T2, five weeks after home confinement), parents reported parental stress, their expressive suppression and adolescents’ anxiety and depression symptoms in response to the quarantine. At Time 3 (T3, end of home confinement, May 2020), parents reported adolescents’ anxiety and depression symptoms in response to the quarantine, and adolescents’ internalizing (both anxiety and depression) symptoms were measured.

### 2.2. Measures

#### 2.2.1. Adolescents’ Anxiety and Depression Symptoms in response to the Quarantine at T1, T2, and T3

Adolescents’ anxiety and depression symptoms in response to the quarantine were measured by the Impact Scale of COVID-19 and home confinement on children and adolescents [1],which measures the immediate psychological effects of COVID-19 on adolescents’ during the quarantine. This scale includes 31 items rated by parents on a 5-point scale, from 1 (*much less compared to before*) to 5 (*much more compared to before*). Only 16 items were used for the current study. Based on previous studies [53,54], we grouped the responses into two categories: anxiety symptoms (10 items; e.g., “is worried” and “is afraid about COVID-19 infection”) and depression symptoms (6 items; e.g., “is sad” and “feels lonely”). In this study, at T1, T2, and T3, the Cronbach’s α were 0.895, 0.790, and 0.748 for anxiety symptoms, and 0.850, 0.755, and 0.736 for depression symptoms, respectively.

#### 2.2.2. Parental Stress at T2

Parental stress was measured by the 10-item Perceived Stress Scale (PSS-10; [17]). Parents were asked about their feelings and thoughts about stressful situations that occurred during the last month to measure the degree to which aspects of their life were unpredictable, uncontrollable, and overloaded. This scale was rated on a 5-point scale (from 0 = *never* to 4 = *very often*), with higher scores indicating greater perceived stress. Sample questions were, “In the last month, how often have you felt that things were going your way?” and “In the last month, how often have you felt that you were on top of things?” The PSS-10 has shown good psychometric properties in Italian samples [18]. In this study, the Cronbach’s α was 0.858.

#### 2.2.3. Parents’ Expressive Suppression at T2

Parents’ expressive suppression was measured by a sub-scale of the Emotion Regulation Questionnaire (ERQ; [34]), which assessed participants’ tendencies to inhibit or reduce the expression of their emotion. The scale includes four items rated on a 7-point scale, from 1 (*strongly disagree*) to 7 (*strongly agree*). Sample questions are, “I keep my emotions to myself” and “I control my emotions by not expressing them”. The ERQ Suppression scale has shown good psychometric properties in Italian samples [55]. In this study, the Cronbach’s α was 0.739.

#### 2.2.4. Adolescents’ Internalizing Symptoms at T3

Adolescents’ internalizing symptoms were measured by two scales which were designed according to the Diagnostic and Statistical Manual of Mental Disorders, to assess anxiety and depression symptoms in adolescents. Anxiety symptoms were measured by the Spence Children’s Anxiety Scale-Parent Version (SCAS-P-8; [56]), which includes eight items and is rated on a 4-point scale (from 0 = *never* to 3 = *always*). Sample questions are, “Worried something bad will happen to me” and “Afraid I will make a fool of myself”. Depression symptoms were measured by the Short Mood and Feelings Questionnaire-Parent Version (SMFQ-P; [57]), which includes 13 items and is rated on a 3-point scale (from 0 = *not true* to 2 = *true*). Sample questions are, “She/he felt miserable or unhappy” and “She/he felt lonely”. Both scales have shown good psychometric properties in Italian samples [31,53]. In this study, the Cronbach’s α was 0.818 for SCAS-P-8, and 0.896 for SMFQ-P.

### 2.3. Data Analysis

The Statistical Package for Social Science (IBM SPSS Version 21) and R Studio (Version 3.6.2) were used for all the data analyses. First, attrition analyses were run to test whether there were differences in the main variables between participants who completed all three surveys (complete group) and participants who dropped out at T1 and/or T2 (attrition group). Second, a repeated measure ANOVA was run to examine how adolescents’ anxiety and depression symptoms to quarantine changed over time. Bonferroni corrections were applied to the *p*-values to reduce the risk of type I errors [58]. Partial eta-squared was used as the effect size, in which small, medium, and large effects were 0.0099, 0.0588, and 0.1379, respectively ([59] p. 283). The cut-off points for the SCAS-P-8 and SMFQ-P were run to examine the levels of adolescents’ anxiety and depression symptoms at T3. Third, descriptive statistics and bivariate correlations were conducted to explore the association between the main variables. We also conducted Pearson correlations, independent t-tests, and ANOVAs to determine potential sociodemographic variables (Table 1) that should be included as covariates in the tests. Finally, Hayes [60] PROCESS Macro (Version 2.13, Model 4) was used to test the mediation analyses. The total effect model was carried out to examine the effects of T2 parental stress on T3 adolescents’ internalizing symptoms without including the mediator. The indirect effect model was conducted to examine the direct and indirect effects of T2 parental stress on T3 adolescents’ internalizing symptoms mediated by T2 expressive suppression. We used 10,000 bootstrap samples from the data [61] and a 95% confidence interval (CI) to determine the significance of the mediating effect. If the CI excluded 0, it indicated a significant mediating effect. Mediation analyses were carried out separately for T3 anxiety symptoms and T3 depression symptoms.

## 3. Results

### 3.1. Attrition Analyses

As shown in Table 1, no significant differences were found in the sociodemographic variables and main variables between the complete group and the attrition group, with the exception of parents’ gender and adolescents’ age.

### 3.2. Adolescents’ Anxiety and Depression Symptoms

Repeated measure ANOVA results showed that adolescents’ anxiety symptoms in response to the quarantine between the three time points were significantly different, with a small effect size (*F*(2, 564) = 4.906, *p* = 0.008, *η_p_*^2^ = 0.017). Post hoc tests showed that anxiety symptoms increased from T1 to T2 (*p* = 0.016) and reduced from T2 to T3 (*p* = 0.017). Regarding adolescents’ depression symptoms to quarantine, the difference between the three time points was statistically different, with a small effect size (*F*(2, 564) = 6.106, *p* = 0.002, *η_p_*^2^ = 0.021). Post hoc tests showed that depression symptoms increased from T1 to T2 (*p* = 0.002), but there was no significant difference at T3.

Based on the proposed cut-off criteria for the SCAS-P-8 (optimal cut-off score of 7.5; [56]) and the SMFQ-P (a mean score of 11 for depressed adolescents; [57,62]), we investigate the percentage of adolescents who scored above the cut-off point. The results showed that a total of 92 adolescents (31.9%) and 51 adolescents (17.7%) presented high anxiety and depression symptoms, respectively.

### 3.3. Descriptive Statistics and Bivariate Correlations

The mean, standard deviations, and bivariate correlations are summarized in Table 2. T2 parental stress was significantly and positively correlated with T2 expressive suppression, T3 adolescents’ anxiety and depression symptoms. T2 expressive suppression was significantly and positively correlated with T3 adolescents’ anxiety and depression symptoms. In general, adolescents’ anxiety and depression in response to quarantine at the three time points were positively correlated with T2 parental stress, T2 expressive suppression, and T3 adolescent’s anxiety and depression symptoms, respectively.

### 3.4. The Effect of Sociodemographic Variables on Expressive Suppression and Adolescents’ Anxiety and Depression Symptoms

Correlations were also used to examine parents’ and adolescents’ age, he mediators, and outcome variables. The results showed that adolescents’ age was negatively correlated with T3 adolescents’ anxiety and depression symptoms. No other significant differences were found. We performed independent t-tests to examine parents’ and adolescents’ gender, the mediators, and outcome variables. The parents’ gender showed significant differences in T3 adolescents’ anxiety (*t*(286) = −3.20, *p* = 0.002, Cohen’s *d =* −0.38) and depression symptoms (*t*(286) = −2.45, *p* = 0.015, Cohen’s *d =* −0.29). No other significant differences were found. ANOVAs were used to examine monthly family income, parents’ education level, mother’s current employment status, father’s current employment status, the mediators, and outcome variables. Parents’ education level had a significant difference in T3 adolescents’ depression symptoms (*F*(3, 284) = 4.98, *p* = 0.002, *η_p_*^2^ = 0.05). Mother’s current employment status had significant differences in T3 adolescents’ anxiety (*F*(6, 281) = 3.00, *p* = 0.007, *η_p_*^2^ = 0.06) and depression symptoms (*F*(6, 281) = 2.86, *p* = 0.010, *η_p_*^2^ = 0.06). Father’s current employment status had a significant difference in T3 adolescents’ anxiety (*F*(5, 274) = 3.21, *p* = 0.008, *η_p_*^2^ = 0.06). No other significant differences were found.

To summarize, when examining the mediation model for T3 adolescents’ anxiety symptoms, adolescents’ age, parents’ gender, mother’s current employment status, and father’s current employment status should be controlled. When examining the mediation model for T3 adolescents’ depression symptoms, adolescents’ age, parents’ gender, parents’ education level, and mother’s current employment status should be controlled. We also controlled for the baseline levels of adolescents’ anxiety and depression symptoms to quarantine at T1 and T2 in the mediation models.

### 3.5. Mediation Analyses

To test the effects of T2 parental stress on T3 adolescents’ internalizing symptoms mediated by the T2 expressive suppression, the mediation analyses were tested by the Hayes PROCESS Macro (Version 2.13, Model 4). Here, we included T3 adolescents’ anxiety symptoms and T3 adolescents’ depression symptoms as outcome variables in the mediation models.

**Figure 1:**
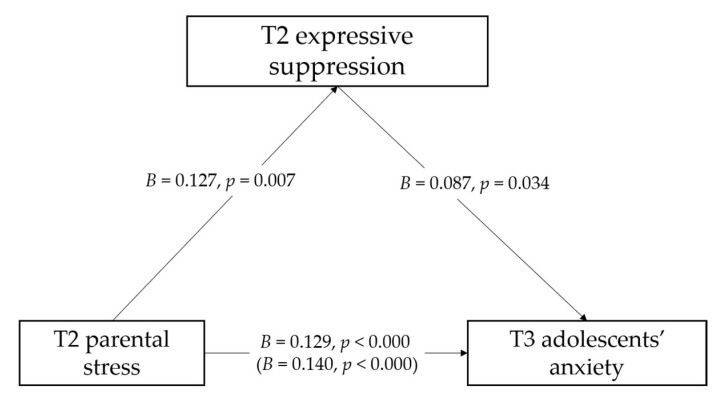
The mediation role of T2 expressive suppression in the association between T2 parental stress and T3 adolescents’ anxiety. Note: The sociodemographic variables and adolescents’ anxiety and depression symptoms to quarantine at T1 and T2 on the mediator and outcome are controlled for but are not shown for simplicity. Slope coefficients are unstandardized. Values in brackets refer to the total effect of T2 parental stress on T3 adolescents’ anxiety without including the mediator.

Referring to T3 adolescents’ anxiety symptoms, the total effect model (Table 3) accounted for 28.3% of variance in the T3 adolescents’ anxiety symptoms. After controlling for the sociodemographic variables and adolescents’ anxiety and depression symptoms to quarantine at T1 and T2, T2 parental stress was positively associated with T3 adolescent’s anxiety symptoms (*B* = 0.140, *SE* = 0.031, *p* = 0.000). The indirect effect model (Table 4 and Figure 1) accounted for 29.5% of variance of T3 adolescents’ anxiety symptoms. After controlling for the sociodemographic variables and adolescents’ anxiety and depression symptoms to quarantine at T1 and T2, T2 parental stress was positively associated with T2 expressive suppression (*B* = 0.127, *SE* = 0.046, *p* = 0.007), as well as T3 adolescents’ anxiety symptoms (*B* = 0.129, *SE* = 0.031, *p* = 0.000). T2 expressive suppression was associated positively with adolescents’ anxiety (*B* = 0.087, *SE* = 0.041, *p* = 0.034). The results of the mediation effects (Table 4) showed that T2 expressive suppression mediated the association between T2 parental stress and T3 adolescents’ anxiety symptoms (*B* = 0.011, *SE* = 0.007, 95% CI = [0.007, 0.190]).

**Figure 2:**
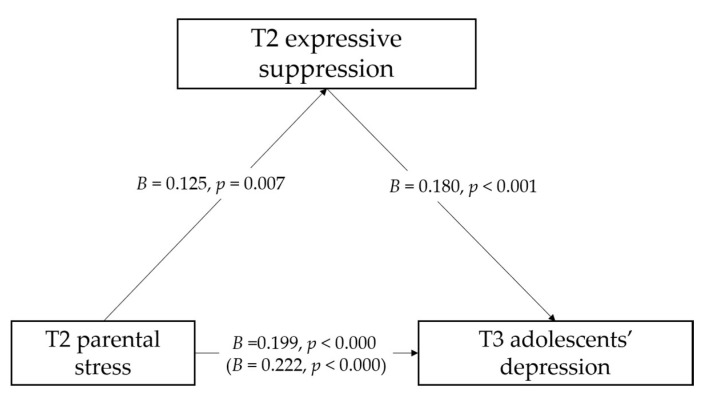
The mediation role of T2 expressive suppression in the association between T2 parental stress and T3 adolescents’ depression. Note: The sociodemographic variables and adolescents’ anxiety and depression symptoms to quarantine at T1 and T2 on the mediator and outcome are controlled for but are not shown for simplicity. Slope coefficients are unstandardized. Values in brackets refer to the total effect of T2 parental stress on T3 adolescents’ depression without including the mediator.

Referring to T3 adolescents’ depression symptoms, the total effect model (Table 3) accounted for 33.2% of variance of T3 adolescents’ depression symptoms. After controlling for the sociodemographic variables and adolescents’ anxiety and depression symptoms to quarantine at T1 and T2, T2 parental stress was positively associated with T3 adolescent’s depression symptoms (*B* = 0.222, *SE* = 0.039, *p* = 0.000). The indirect effect model (Table 4 and Figure 2) accounted for 36.3% of variance of T3 adolescents’ depression symptoms. After controlling for the sociodemographic variables and adolescents’ anxiety and depression symptoms to quarantine at T1 and T2, T2 parental stress was positively associated with T2 expressive suppression (*B* = 0.125, *SE* = 0.046, *p* = 0.007), as well as T3 adolescents’ depression symptoms (*B* = 0.199, *SE* = 0.038, *p* = 0.000). T2 expressive suppression was positively associated with adolescents’ depression symptoms (*B* = 0.180, *SE* = 0.050, *p* = 0.000). The results of the mediation effects (Table 4) showed that T2 expressive suppression mediated the association between T2 parental stress and T3 adolescents’ depression symptoms (*B* = 0.023, *SE* = 0.011, 95% CI = [0.006, 0.050]).

### 3.6. Supplementary Analyses

Considering that the independent variable and mediator were measured at the same time, we also tested the competing models that switched the independent variable and the mediator (i.e., T2 expressive suppression was the new independent variable and T2 parental stress was the new mediator). As shown in Table 5, the results suggested that the models initially hypothesized were the best fit compared to the competing model, in terms of lower values of Akaike Information Criterion (AIC) and the Bayesian Information Criterions (BIC).

## 4. Discussion

COVID-19 and its restrictions have had long-term and profound impacts on adolescents. Moreover, in the short term, COVID-19 will not disappear from our daily lives and will remain a challenge to adolescents in terms of their mental health [1,2,3,4]. This study adopted a longitudinal design for three consecutive time periods to investigate changes in anxiety and depression symptoms of Italian adolescents during COVID-19 and its restrictions. At the same time, based on emotion regulation theory, we explored the predictive effect of parental stress on adolescents’ anxiety and depression symptoms, and the mediating effect of expressive suppression.

The results confirmed our hypothesis and showed that after the implementation of home confinement, adolescents’ anxiety and depression symptoms changed significantly over time. Specifically, compared with those at two weeks after home confinement, adolescents’ anxiety and depression symptoms were significantly increased at five weeks, which was consistent with previous studies [10,11,12,13]. Our data suggest that exacerbation of internalizing symptoms did not occur at the start of home confinement but during the longer confinement period. Home confinement and the related measures have stronger impacts on mental health than fear of the new virus itself. Our results also prove that home confinement is a risk factor for adolescents’ internalizing symptoms and are consistent with the findings of Brooks et al. [2] that extended quarantine is a predictor of psychological distress. In addition, adolescence represents the stage of gradual independence from the family and the establishment of new emotional support and social development through peer interactions [63]. However, COVID-19 and its restrictions require adolescents to stay at home. Maintaining social distance from others hinders adolescents’ interactions with their peers and reduces their social contact, which may lead to higher levels of anxiety and depression symptoms. The importance of social connections is well known; for example, previous studies have found that home confinement during COVID-19 can induce loneliness and social isolation stress, which are associated with internalizing symptoms [64,65]. Moreover, the longitudinal change of internalizing symptoms may also be linked to how the Italian government managed the home confinement. In the beginning, adolescents were sufficiently resilient to respond to the stressful situation [66]. However, when the home confinement was extended each week, they would lose hope and managed the difficulties faced in less adaptive ways [2].

We also found that at the end of home confinement compared with those at five weeks, adolescent’s anxiety was decreased, but depression symptoms were moderately stable without significant longitudinal changes, which was similar to the results of studies in China [14], Spain [15], and the United States [67]. Adolescents’ anxiety symptoms were reduced, which may be because the end of the quarantine period meant that the threat of COVID-19 to life was reduced, and fears were lessened, possibly due to the removal of the strict measures put in place. Moreover, adolescents could go out, providing opportunities for social interactions. This suggests that anxiety is more related to the COVID-19 situation and home confinement, and anxiety showed a decreasing trend when the rules ended. However, depression symptoms were the same as those at five weeks after the home confinement, possibly because the schools did not reopen at the end of the home confinement, and the opportunities for social interactions through schools were still banned. Previous studies have shown that the long-term harmful effects of COVID-19 are likely to be magnified by further school closures [13,67]. Our results indicate that end of home confinement does not mean a decrease of all symptoms and that depression symptoms (i.e., loneliness and social isolation stress) may have stronger and longer impacts on mental health. In addition, the results showed that at the end of home confinement, 31.9% of adolescents experienced higher levels of anxiety symptoms, and 17.7% of adolescents experienced higher levels of depression symptoms. This illustrates the need to focus on adolescents’ anxiety and depression after home confinement to prevent a deterioration of their internalizing symptoms.

Second, we found that high levels of parental stress significantly predicted increased anxiety and depression symptoms in adolescents during quarantine, which was consistent with previous studies [31,32]. It suggests that even in adolescence, parental factors play an important role in shaping the emotions and behaviors of adolescents. The pandemic and its restrictions have increased the time that parents spend with their children, and parents have become the closest contacts for their children. The relationship between the emotional state experienced by parents during the public health emergency and their children’s emotional states has been confirmed [9,30]. The current longitudinal study confirms that parental stress can predict the possibility of adolescents’ experiencing anxiety and depression symptoms and provides evidence that higher levels of parental stress may be a key risk factor leading to internalizing symptoms in adolescents during crises [27,28].

More importantly, we confirmed the mediating role of expressive suppression in the association between parental stress and adolescents’ internalizing symptoms, which supported the emotion regulation theory [34,37]. During the quarantine, parents have been stressed at work and at home [19]. The daily issues of the parent–child relationship may become more difficult due to spending more time together [1,68]. The measures making people stay at home have resulted in parents losing the space and time for self-regulation [69]. They not only need to regulate their own emotions but also need to calm the emotional state of their children [27,70,71]. This may be why parents are more inclined to use the emotion regulation strategy of expressive suppression, as it can help them to cover up their negative emotions and avoid showing negative emotional states that would make other family members feel anxious. However, in public health emergencies, parents have become the most important social reference templates for children and adolescents [47,72]. Parents’ avoidance and suppression of stress will be imitated by their children. This may cause adolescents to suppress their emotional expressions, resulting in a build-up of their negative emotions. The longer the duration, the more likely they are to develop internalizing symptoms. The association between the use of expressive suppression and internalizing symptoms in adolescents has been confirmed [73]. The current study provides additional evidence that parents’ expressive suppression would also increase the probability of adolescents’ anxiety and depression symptoms. On the other hand, parents are one of the main sources of information and social connections for children and adolescents during the quarantine [71]. If parents suppress their emotional expression, adolescents cannot accurately obtain the same information, nor can they gain a sense of security from their parents. Previous studies have shown that when children and their parents experience a stressful situation if the parents’ ability to convey a sense of safety to their children is debilitated, this may magnify the difficulties for children’s emotional regulation [32,74,75].

To the best of our knowledge, few studies exist on longitudinal changes in adolescents’ anxiety and depression symptoms during COVID-19 and the ensuing restrictions. The current study bridges this gap and shows that the quarantine period is a risk factor for adolescents’ internalizing symptoms. This study provides additional information in terms of the emotion regulation theory and highlights the important role of parents for adolescents during major disasters or public health emergencies. We showed that during a crisis, high levels of parental stress would increase adolescents’ internalizing symptoms via parental expressive suppression. These findings suggest that we should take note of the changes in adolescents’ emotions. The end of the home confinement does not mean the end of anxiety and depression symptoms; adolescents remain in a precarious situation, and they will need long-term input and psychological support after the quarantine ends. Second, parents’ effective emotional regulation strategies can reduce their own negative emotions and help their children’s emotion regulation. Therefore, more support should be given to help parents cope with the home confinement measures during a pandemic. At the same time, parents can strengthen the parent–child relationship through active parent–child interactions and coping strategies and give their children support and a sense of safety, such as active discussions about the epidemic [26] and sharing their emotions [50]. Third, the government and public health can provide active assistance measures, such as online psychological counseling, to provide psychological guidance and intervention for parents and young people.

This study has some limitations. First, due to the limitations imposed by COVID-19, this study took the form of an online survey that only reported information from parents. Even if the parents are the most concerned about adolescents during the quarantine period, it is strongly recommended that future studies adopt multiple evaluation methods (e.g., adolescents’ self-reporting, interviews, and observations) to be more objective. Second, we conducted the follow-up investigations three times during the first COVID-19 quarantine in Italy between March and May 2020. However, the virus is constantly mutating, and it is uncertain whether the government will implement home confinement measures again. Adolescents’ mental health recovery still needs long-term exploration. Third, this study only focused on expressive suppression due to its close relation to internalizing symptoms; however, cognitive reappraisal, which represents another emotion regulation strategy, should also be considered. Previous studies have found that the use of cognitive reappraisal is related to greater positive emotions [76]. Future studies can investigate whether cognitive reappraisal has a positive effect on the mental health of adolescents after home confinement. Despite these limitations, this study provides information on the risks of prolonged home confinement for the mental health of adolescents and their parents. Moreover, it provides evidence and references for crisis management during public health emergencies, and especially for interventions targeting the family environment.

## 5. Conclusions

During the period of COVID-19 and its restrictions, adolescents may encounter various difficulties and challenges that impact their mental health. The current study found that long-term home confinement increases adolescents’ internalizing symptoms. Moreover, high levels of parental stress due to COVID-19 predict an increase in anxiety and depression symptoms of adolescents via the use of parents’ expressive suppression. We suggest that after home confinement ends, attention should be focused on the changes in adolescent mental health, the mental health of their parents, and the underlying mechanisms of interactions between them. The entire family should be helped to recover through active interventions and assistance, and further studies should explore positive coping strategies to provide psychological guidance for parents and adolescents in responding to public health emergencies.

## Figures and Tables

**Table 1:** Attrition analysis.

Variables	Complete Group (*n* = 288)		Attrition Group (*n* = 763)		Test ^a^
	*M*	*SD*	*M*	*SD*	
T1 adolescents’ anxiety to quarantine	2.91	2.46	2.85	2.52	0.392
T2 adolescents’ anxiety to quarantine	3.31	2.44	2.90	2.30	1.797
T1 adolescents’ depression to quarantine	2.18	1.73	2.08	1.67	0.829
T2 adolescents’ depression to quarantine	2.57	1.71	2.59	1.67	−0.090
T2 parental stress	19.02	7.18	18.78	7.64	0.339
T2 expressive suppression	13.17	5.11	12.73	4.44	0.919
Parents’ age	46.50	4.65	46.39	5.72	0.274
Adolescent’s age	13.89	2.15	14.22	2.29	−2.114 *
	*N*	*%*	*N*	*%*	
Parents’ gender (female)	275	95.5	653	85.6	19.842 ***
Monthly family income (euros)					3.136
Up to 999	4	4.5	9	3.8	
Between 1000 and 1999	18	20.2	65	27.1	
Between 2000 and 3999	29	32.6	60	25.0	
Between 3000 and 4999	27	30.3	81	33.8	
5000 or more	11	12.4	25	10.4	
Education level					1.017
Primary school	21	7.3	70	9.2	
Secondary school	133	46.2	339	44.5	
Undergraduate	94	32.6	245	32.2	
Doctoral or master	40	13.9	107	14.1	
Mother’s current employment situation					8.666
Self-employed	40	13.9	92	12.2	
Part-time	73	25.3	178	23.5	
Full-time	59	20.5	200	26.5	
Unemployed	20	6.9	45	6.0	
Lost job due to COVID-19	19	6.6	27	3.6	
Home-working	54	18.8	147	19.4	
Other	23	8.0	67	8.9	
Father’s current employment situation					8.706
Self-employed	76	27.1	214	28.7	
Part-time	13	4.6	45	6.0	
Full-time	133	47.5	326	43.7	
Unemployed	8	2.9	25	3.4	
Lost job due to COVID-19	8	2.9	16	2.1	
Home-working	42.0	15.0	103.0	13.8	
Other	0	0	17.0	2.3	
Adolescents’ gender (female)	130.0	45.1	386.0	50.6	2.486

Notes: * *p* < 0.05, *** *p* < 0.001. ^a^ Cross-table (χ^2^) for categorical variables and independent *t*-test for continuous variables.

**Table 2:** Means, standard deviations, and bivariate correlations of the main variables (*n* = 288).

	1	2	3	4	5	6	7	8	9	10	11	12
1. Parent age	–											
2. Adolescent age	0.31 **	–										
3. T1 adolescents’ anxiety to quarantine	0.06	−0.04	–									
4. T2 adolescents’ anxiety to quarantine	−0.01	−0.02	0.57 **	–								
5. T3 adolescents’ anxiety to quarantine	−0.02	−0.08	0.46 **	0.56 **	–							
6. T1 adolescents’ depression to quarantine	0.04	−0.03	0.61 **	0.35 **	0.31 **	–						
7. T2 adolescents’ depression to quarantine	0.01	−0.14 *	0.40 **	0.54 **	0.34 **	0.49 **	–					
8. T3 adolescents’ depression to quarantine	−0.04	−0.15 *	0.40 **	0.50 **	0.62 **	0.39 **	0.58 **	–				
9. T2 parental stress	−0.07	−0.05	0.27 **	0.27 **	0.25 **	0.19 **	0.22 **	0.20 **	–			
10. T2 expressive suppression	0.06	−0.01	0.13 *	0.15 *	0.18 **	0.09	0.05	0.17 **	0.17 **	–		
11. T3 adolescents’ anxiety	−0.04	−0.19 **	0.38 **	0.28 **	0.42 **	0.31 ***	0.20 **	0.35 **	0.35 **	0.23 **	–	
12. T3 adolescents’ depression	−0.01	−0.13 *	0.34 **	0.36 **	0.51 **	0.33 **	0.40 **	0.60 **	0.41 **	0.27 **	0.67 **	–
*M*	46.50	13.89	2.91	3.31	2.93	2.18	2.57	2.36	19.02	13.17	6.26	5.94
*SD*	4.65	2.15	2.47	2.44	230	1.73	1.70	1.73	7.18	5.11	3.92	5.16

Notes: * *p* < 0.05, ** *p* < 0.01.

**Table 3:** The total effect of each pathway in the models (*n* = 288).

Paths	*B*	*SE*	*p*
**DV: T3 Adolescents’ Anxiety (*R*^2^ = 0.283)**			
T2 parental stress → T3 adolescents’ anxiety	0.140	0.031	<0.000
T1 adolescents’ anxiety to quarantine → T3 adolescents’ anxiety	0.365	0.120	0.003
T2 adolescents’ anxiety to quarantine → T3 adolescents’ anxiety	0.115	0.111	0.303
T1 adolescents’ depression to quarantine → T3 adolescents’ anxiety	0.221	0.161	0.171
T2 adolescents’ depression to quarantine → T3 adolescents’ anxiety	−0.114	0.156	0.465
adolescent’s age → T3 adolescents’ anxiety	−0.311	0.097	0.002
parents’ gender → T3 adolescents’ anxiety	1.152	0.991	0.246
mother’s current employment status → T3 adolescents’ anxiety	−0.051	0.106	0.630
father’s current employment status → T3 adolescents’ anxiety	0.153	0.128	0.233
**DV: T3 adolescents’ depression (*R*^2^ = 0.332)**			
T2 parental stress → T3 adolescents’ depression	0.222	0.039	<0.000
T1 adolescents’ anxiety to quarantine → T3 adolescents’ depression	0.117	0.148	0.432
T2 adolescents’ anxiety to quarantine → T3 adolescents’ depression	0.245	0.140	0.082
T1 adolescents’ depression to quarantine → T3 adolescents’ depression	0.336	0.201	0.096
T2 adolescents’ depression to quarantine → T3 adolescents’ depression	0.472	0.195	0.016
adolescent’s age → T3 adolescents’ depression	−0.223	0.122	0.069
parents’ gender → T3 adolescents’ depression	−0.185	1.273	0.885
parents’ education level → T3 adolescents’ depression	−0.933	0.321	0.004
mother’s current employment status → T3 adolescents’ depression	−0.081	0.132	0.540

**Table 4:** The specific direct and indirect effects for each pathway in the models (*n* = 288).

Paths	Direct Effects			Indirect Effects		
	*B*	*SE*	*p*	*B*	*SE*	95% Bootstrapping CI
**DV: T3 Adolescents’ Anxiety**						
T2 parental stress →T2 expressive suppression	0.127	0.046	0.007			
T2 parental stress →T3 adolescents’ anxiety	0.129	0.031	<0.000			
T2 expressive suppression → T3 adolescents’ anxiety	0.087	0.041	0.034			
T2 parental stress→ T2 expressive suppression →T3 adolescents’ anxiety				0.011	0.007	[0.001, 0.032]
**DV: T3 Adolescents’ Depression**						
T2 parental stress →T2 expressive suppression	0.125	0.046	0.007			
T2 parental stress → T3 adolescents’ depression	0.199	0.038	<0.000			
T2 expressive suppression → T3 adolescents’ depression	0.180	0.050	<0.001			
T2 parental stress→ T2 expressive suppression →T3 adolescents’ depression				0.023	0.011	[0.006, 0.050]

**Table 5:** Summary of the model fit indices of the competing models.

	Model Fit Indices								
	AIC	BIC	*χ* ^2^	*df*	CFI	TLI	RMSEA	90%CI	SRMR
Hypothesized model for depression symptoms	3343.42	3390.82	9.01	8	0.992	0.981	0.021	[0.000, 0.075]	0.023
Competing model for depression symptoms (switch X and M)	3534.66	3582.05	51.47	8	0.743	0.390	0.139	[0.104, 0.176]	0.070
Hypothesized model for anxiety symptoms	3122.18	3169.20	9.66	8	0.983	0.959	0.027	[0.000, 0.079]	0.026
Competing model for anxiety symptoms (switch X and M)	3311.49	3358.51	51.24	8	0.687	0.257	0.140	[0.105, 0.178]	0.070

Notes: AIC = Akaike Information Criterion; BIC = Bayesian Information Criterions; CFI = Comparative Fit Index; TLI = Tucker–Lewis index; RMSEA = Root Mean Square Error of Approximation; SRMR = Standardized Root Mean Square Residual.

## Data Availability

The data presented in this study are available on request from the corresponding author.
